# Towards biology-informed therapies for long COVID

**DOI:** 10.1016/j.ebiom.2026.106302

**Published:** 2026-05-13

**Authors:** eBioMedicine

The US Centers for Disease Control and Prevention estimates between 2.8% and 4.4% of adults in the USA are currently living with post-acute sequelae of COVID-19 (PASC; or long COVID) and that between 1.7% and 2.9% of US adults face substantial limitations in day-to-day activities due to PASC. Yet, despite hundreds of registered interventional trials aimed at addressing PASC, as of May, 2026, we still lack approved and broadly effective disease-modifying therapies for this debilitating condition.

The slow pace of progress in PASC research is probably partly attributable to the marked heterogeneity of the disease, with symptoms varying widely in nature and intensity both within and between individuals. These symptoms in turn are probably the result of one or several potential biological aetiologies, further complicating the identification of viable therapeutic targets.

How do we find and test treatments for such a complex disease? As outlined in a personal view from Julia Vogel and colleagues published in the January, 2026 issue of *eBioMedicine*, the answer is likely to involve, in part, stratification of patients based on pathobiology. But to get there, we first need to better understand the mechanisms underpinning the heterogeneous symptoms of PASC, and to identify biomarkers that will allow us to both stratify and treat based on the underlying biology. With these aims in mind, researchers have been focusing efforts on at least three lead concepts: the role of autoantibodies, immune dysregulation, and SARS-CoV-2 viral persistence.

A systematic review published in the April, 2026 issue of *The Lancet Infectious Diseases* examined the relationship between autoantibodies and PASC. In the 44 studies that met the inclusion criteria, including a total of 3372 individuals with long COVID, 31 (71%) of the studies reported associations between autoantibodies and PASC symptoms. The main autoantibody types identified as potential biomarkers included antinuclear antibodies, autoantibodies against G protein-coupled receptors, and autoantibodies against chemokines. These autoantibodies were able to distinguish between people with PASC and comparator groups, and tracked with symptom severity in some cases. However, as noted by the authors, an important caveat is the highly heterogeneous nature of the evidence—with varying definitions of long COVID, variable timing of antibody measurements, and small sample sizes. Given these limitations, larger, well controlled studies are needed to confirm whether autoantibodies could be reliable biomarkers of disease, and whether they might play a causative role in PASC symptoms.

Mounting evidence also suggests that immune dysregulation may also be a common feature in some people with long COVID. A multi-omics immune profiling study by Barouch and colleagues (*Nature Immunology*, December, 2025) and a plasma proteomics analysis by Dahl and colleagues (*Frontiers in Immunology*, February, 2026) both report associations between PASC and ongoing inflammatory signalling. Interestingly, both studies found persistent immune activation of interleukin-6 and JAK-STAT-related pathways. However, it is important to note that these findings are based on group-level differences and are not yet established as patient-level biomarkers. Although Barouch and colleagues tested for plasma viral RNA, and Dahl and colleagues for circulating viral spike protein, it is worth mentioning that neither found evidence of persistent viral replication in patients with long COVID compared with recovered controls.

Although detection of replication-competent virus in patients with long COVID has proven to be technically challenging, the idea that a persistent SARS-CoV-2 infection or so-called viral reservoir might contribute to pathology remains a viable hypothesis. Two clinical trials (PAX LC published in *The Lancet Infectious Diseases*, August, 2025; and STOP-PASC published in *JAMA Internal Medicine*, June, 2024) tested this hypothesis by administering the antiviral nirmatrelvir-ritonavir for 15 days to people with long COVID symptoms. Unfortunately, neither study saw improved health outcomes in participants given the antiviral, compared with placebo-ritonavir, when assessed at day 28. However, the treatment duration was fairly short, and it is possible that there could be tissue-based viral reservoirs that are difficult to target effectively. Given that both studies did not have validated biomarkers to identify and monitor viral persistence, the possibility remains that a biologically defined subgroup of PASC patients could benefit from a targeted antiviral approach.

Several plausible mechanisms to explain long COVID pathology have been proposed. The next phase of progress will require coupling these biological mechanisms to reproducible, validated biomarkers to guide patient selection and help measure biologically meaningful outcomes.

